# Pandemic preparedness: synthetic biology and publicly funded biofoundries can rapidly accelerate response time

**DOI:** 10.1038/s41467-022-28103-3

**Published:** 2022-01-21

**Authors:** Claudia E. Vickers, Paul S. Freemont

**Affiliations:** 1 CSIRO Synthetic Biology Future Science Platform, CSIRO Land & Water, EcoSciences Precinct, Dutton Park, 4012 Australia; 2 ARC Centre of Excellence in Synthetic Biology, Queensland University of Technology, Brisbane, 4000 Australia; 3 Griffith Institute for Drug Design, Griffith University, Nathan, 4111 Australia; 4 Section of Structural and Synthetic Biology, Department of Infectious Disease, Imperial College London, Sir Alexander Fleming Building, South Kensington Campus, South Kensington, London, SW7 2AZ UK; 5 UK Dementia Research Institute Care Research and Technology Centre, Imperial College London, Hammersmith Campus, Du Cane Road, London, W12 0NN UK; 6 UK Innovation and Knowledge Centre for Synthetic Biology (SynbiCITE) and the London Biofoundry, Imperial College Translation & Innovation Hub, White City Campus 80 Wood Lane, London, W12 0BZ UK

**Keywords:** Synthetic biology, Policy and public health in microbiology

## Abstract

Synthetic biology has played a key role in responding to the current pandemic. Biofoundries are critical synthetic biology infrastructure which should be available to all nations as a part of their independent bioengineering, biosecurity, and countermeasure response systems.

## Introduction: the current context

**In recent years, we have seen outbreaks of SARS, MERS and Ebola; and we are currently dealing with the SARS-CoV-2 pandemic. This pandemic has demonstrated the power of biology, both in our vulnerability to it and in our ability to engineer solutions to global problems using it. The remarkable feat of bringing novel, safe, effective vaccines to the market inside a 12-month window from when the coronavirus was identified shows that we can move this technology very quickly when the need is critical. Nonetheless, as of writing, we have lost almost 4.8 million people across the world – a number which is rapidly increasing**^1^ **– and global vaccine roll-out has a very long way to go. The frequency of emerging pandemic-capable diseases is increasing**^2^, **and our ability to respond must also accelerate to prevent large-scale loss of life. The learned experience of the current crises is that the global nature of pandemics requires a global response if we are to minimise loss of life and economic disruption.**

In bioengineering, there is a limit to the number of DNA-encoded solution candidates that can be tested due to bottlenecks in assembly of DNA componentry where throughput is determined by the number of hands available. Synthetic biology can accelerate this approach. In particular, the use of Biofoundries – high-throughput robotic DNA and organism engineering facilities^3^ – can generate hundreds or thousands of constructs/strains in just a few days. Coupled with standardised DNA componentry, high-throughput screening systems, iterative engineering through design-build-test-learn cycles, and machine learning algorithms to interrogate the data and suggest design options, this provides the opportunity to sample a much greater bio-design space much more rapidly. This scale of exploration holds the promise to identify more, and potentially better, solutions to a given problem. These characteristics make biofoundries critical for rapid countermeasure responses to emerging threats such as pandemics, as well as locally emerging pests, pathogens and variants thereof. However, access to biofoundries is currently limited to a few wealthy nations, and the technology is still relatively nascent, with more development required to deliver on their full potential.

## Where biofoundaries can contribute

There are numerous pandemic countermeasures to which biofoundries can contribute developmental capability. Vaccine development is perhaps the most obvious. Much of the time expenditure between identification of a new or variant virus and delivery of a vaccine is due to factors such as scale-up of production, the regulatory and policy environment, coordination and distribution issues, and sociopolitical and geopolitical issues^4–11^. However, vaccine design remains challenging, with significant trial-and error; and the speed of vaccine development remains a bottleneck in the delivery pipeline^4–6,11^. Moreover, for local emerging diseases (including humans, plants and animals), an independent ability to respond rapidly is absent in many countries. To respond to new variants and novel emerging infectious diseases, there is a need for bioengineers to much more rapidly prototype vaccine and diagnostics candidates. New technologies such as the RNA vaccines now available are particularly amenable to this approach, but it is also very applicable to more ‘classical’ vaccines, including protein-based vaccines; inactivated viral capsids, synthetic viral capsids and sVLP decorated with target antigens. Biofoundries can also be used for high-throughput serological testing of vaccines and to accelerate development of new adjuvants for vaccine delivery. In combination with distributed manufacturing to deliver small-scale broadly available manufacturing, biofoundries offer the potential to revolutionise vaccine delivery^12^.

The development of diagnostics tools can also be accelerated using biofoundries. For example, paper-based point-of-care kits require high-throughput screening of protein/peptide antigen variants using large libraries, an approach that can be achieved at much higher rates using fluid-handling robots^13,14^. Therapeutics development can also be accelerated, again by applying high-throughput approaches. Applications include testing of therapeutic antibodies and recombinant antigens, small molecule immunomodulators, and antiviral drugs (e.g., protease inhibitors). Combining diagnostics tools with therapeutic delivery for theranostics development required particularly high combinatorial matrices for acceleration, something that biofoundries are adept at delivering^15,16^.

In addition to accelerating the development of vaccines, diagnostics, and therapeutics, the liquid-handing robots in biofoundries are ideal for high-throughput infection testing using PCR or serological approaches. This means that a biofoundry can rapidly be converted to an independent test facility^17^ and contribute to the epidemiological analysis. As pandemics progress, variant surveillance becomes critically important - particularly where variants have increased transmissibility or where vaccine effectiveness is decreased.

## Challenges and lessons learned

Biofoundries allow us to explore science and bioengineering solution spaces on a much greater scale than previously possible, which increases the opportunity to rapidly develop pandemic response solutions and deliver them to the market. However, as an emerging technology, we are still learning how to best use biofoundries; exploration on this scale requires a quite different operational and design of experiments (DOE) approach than classical investigations. The Global Biofoundries Alliance (GBA)^18^ is a consortium of academic biofoundries founded on principles of open sharing of resources and data to help accelerate biofoundry development worldwide. Cooperation through the GBA has allowed some countries which were relatively late starters (such as Australia) to leapfrog to the cutting edge of this technology. Lessons learned have been published so they are available for other countries that wish to establish their own biofoundries^3^. As the pandemic emerged in early 2020, several biofoundries sought to contribute to global efforts to combat the disease. The GBA facilitated rapid sharing of information and methodologies during this time. Key learnings included:

- Awareness about the capabilities of biofoundries is poor outside the GBA. Increasing awareness of what biofoundries can do and supporting countries to develop their independent capability is needed.
- Biofoundries must work within existing diagnostics and vaccine development frameworks including regulatory systems to contribute to a broader effort. This means plugging into existing processes (which are typically closed systems rather than the open and flexible platforms used in biofoundries) instead of developing new processes (Box 1: BioFoundry Pivot Case Studies)
- Technologies can be rapidly reported and shared between biofoundries (Box 1: Biofoundry Pivot Case Studies), providing the opportunity for countries to support each other in countermeasure responses. This is critical, because in a pandemic situation, no country is ‘safe’ until every country is safe.
- Unexpected technological, regulatory, and sociological hurdles prevented some biofoundries from engaging effectively. For example, getting regulatory approval to operate as a diagnostic testing facility requires an extremely high level of accreditation (e.g. ISO 15189). Prior accreditation could allow for much more rapid conversion in a countermeasure scenario.
- Acute supply chain issues affected reagent availability, crippling the ability of some biofoundries to contribute to the response. Ensuring that reagents are available when and where needed for maximum response efficiency (testing and vaccine development) is therefore critical.

Box 1 BioFoundry Pivot Case Studies In March 2020, the total SARS-CoV-2 testing capacity in the world was extremely limited (e.g. in the UK the national capacity was ~10,000 tests a day). This lack of preparedness in both diagnostic and asymptomatic testing led to a global scramble for instrumentation and reagents where national governments were competing to secure their supply chain needs. The delay in rolling out widespread global testing inevitably led to increased transmission and subsequent deaths. In response to the pandemic emergency, several biofoundries quickly pivoted their expertise and in infrastructure to provide increased testing, two of which are illustrated below. It should be noted that other approaches have also been successful including the Shield saliva testing program at the University of Illinois (https://shieldillinois.com/) and the impressive Concentric program by Gingko Bioworks for school testing (https://www.concentricbyginkgo.com/).

*London Biofoundry at Imperial College London, UK*. Unlike other countries, the UK adopted a two-pillar system where large ‘Amazon-like’ testing labs were established from scratch for community testing whilst existing National Health Service (NHS) pathology labs were enhanced for hospital testing. In late February 2020, the London Biofoundry (LBF) pivoted to develop an automated modular open PCR-testing platform that was reagent agnostic, scalable, and could carry out 1000 tests per day. By working closely with HNS diagnostic specialists, in late April the London Biofoundry platforms were quickly implemented and UKAS accredited for front line patient testing at both Charing Cross and St Mary’s hospitals in North West London. Early this year a community testing ‘Lighthouse Lab’ was established, delivering 3,000 tests a day to the UK government’s Department of Health and Social Care. The open source and modular design of the platforms have now been adopted for both NHS Imperial Trust hospitals and staff, student and community testing at Imperial college and by October 2021, over 800,000 tests have been carried out using the LBF’s platform design and workflow. The LBF also developed an open-source synthetic virus-like particle based on bacteriophage MS2, which could be used as a safe Cat-1 testing control. This reference material is now part of a Coronavirus Standards Consortium study on RNA PCR controls^19^. Given the importance of variant surveillance, the LBF has also established a Nextgen high-throughput sequencing workflow which has carried out sequencing on all positive tests from Imperial college staff and students.

*DAMP Lab at Boston University, USA*. The DAMP lab was established by Prof. Douglas Densmore as a biofoundry that aims to develop novel biological systems using formal representations of protocols and experiments for the synthetic biology “design-build-test” cycle^20^. Early in the pandemic, Boston University (BU) developed an ambitious testing plan that would enable 45,000 tests routinely with up to 6000 tests a day. As part of this effort, the DAMP lab (working with BU’s Precision Diagnostic Centre) established and implemented all the necessary robotic and software automation for the testing programme which comprised as many as eight large-scale liquid handling robots and five qPCR machines^21^. The net result is that BU has been able to offer a comprehensive testing programme to its community, and since July 2020 has completed over 1 million SARS-CoV-2 tests with an average processing time of <24 h. The speed of rolling out this service as provided by the DAMP Lab clearly illustrates the power of having localised biofoundry expertise and testing infrastructure.

## The future: democratisation and rapid response delivery

An equity problem with biofoundries is the high cost of establishment, ongoing operational expenses, and access fees^3^, making it challenging for many countries to have their own biofoundries. Moreover, supply chain disruption in a pandemic, as well as general local disruption, impacts on research and facility operations. Affordable regional biofoundry access and/or exploitation of the global biofoundry network might at least partly help address this problem. A perspective detailing the technical and operational considerations for establishing a biofoundry has recently been published^3^ and can help guide establishment of facilities. A Biofoundry would need to be established in advance of a pandemic and used for broader research and development, so that it is available to pivot in the case of a local outbreak of a dangerous disease or a global pandemic. This would allow timely local testing capability in addition to diagnostics/therapeutics development, depending on the need. The GBA is focused on open-source technologies and sharing of data and information to accelerate the development of biofoundry technology. However, much technology is held in the private domain and protected through patents and other mechanisms. Licensing agreements that allow biofoundries to modify existing technologies for application to local needs or use at reduced cost could help accelerate the development of response technologies. Furthermore, the world’s largest biofoundry (Gingko Bioworks) is in the private sector, illustrating the technological benefits of the biofoundry approach. Whilst Gingko has made huge and successful efforts in supporting the pandemic response in the US, a long-term democratisation of biofoundries is required to provide equitable access across the globe.

Going into the future, democratisation of BioFoundry technology – especially, making this technology and its products accessible to developing countries – will be an essential part of delivering global pandemic preparedness. To be pandemic-ready, countries and biofoundries must work together and with other key global organisations (e.g., WHO, CEPI, OECD, World Bank, philanthropic foundations, GBA, etc.), continue to develop and improve biofoundry technologies, and prepare effectively (see Box 2). Governments and funding agencies across the world must recognise the importance of this emerging technology not only for their ability to deliver novel science and engineering solutions, but also for their ability to deliver independent bioengineering, biosecurity, and countermeasure capabilities as well as being part of a global infrastructure/capability response to future pandemics. As shown in the current crises, a network of publicly funded biofoundries can pivot quickly and provide impactful and coordinated solutions to enhance national emergency responses. However, to do so, such capabilities need to be established and funded over the long term and become part of national critical infrastructure to ensure that they are available when they are needed. A closer interaction between biofoundries and companies that deliver diagnostics, vaccines, therapeutics, and testing capabilities will be needed to exploit the potential that biofoundries have to accelerate delivery pipelines. Multilateral support to pressure test infrastructure networks for preparedness will be important learning opportunities to ensure that we are better prepared globally to deliver a faster response for the next pandemic.

Box 2 How biofoundries can prepare for rapid countermeasures

1. *Prepare in advance*. Embed learnings from the current experience into operational strategy, for example: increase awareness of biofoundry capabilities; work within existing frameworks; obtain relevant accreditations; engage with regulatory policy; have distributed supply chains and/or develop local low-cost reagent manufacturing (e.g., off-patent reagents). Participate in exercises designed to improve preparedness and ensure that workflows delivering pandemic preparedness pipelines are effectively established.
2. *Broad engagement with policy development and the public* is critical. The adoption of new biotechnologies necessitates dialogues with multiple stakeholders, particularly in countries where biotechnology and synthetic biology are not well understood or does not form a major part of the national/regional economy. Biofoundries must also meet local and international regulatory standards and be prepared to actively engage with appropriate regulators to achieve this.
3. *Support global commons development* and open sharing of data and technologies so that platforms developed in one biofoundry can be rapidly ported to other biofoundries. Effective coordination across a global virtual platform of infrastructure and expertise requires good communication and consistency between measurement approaches and data types (metrology). Engage effectively with global organisations that support development and share information – for example, the Global Biofoundries Alliance.
4. *Support access to biofoundries and/or development of biofoundries* in other countries. Equity and access are critical to rapid responses and for overall development of these technologies. This has been recognised and is currently being examined as part of a broader investigation into synthetic biology through the World Economic Forum Global Future Council in Synthetic Biology^22^. In their first blog post^23^, the Council identifies a need to centre values of equity, sustainability, solidarity and humility in order to realise the true potential of synthetic biology to benefit people and the planet.
5. *Coordinate effectively* and support both local and international responses. When pandemics emerge, the ability to do science is often fragmented because of breakdown of supply chains and broader outbreak impacts such as travel bans leading to transportation problems. Cloud-based access, open data and 24/7 parallelised global research and response will massively accelerate discovery and development rates.
6. *Support the shift in paradigm* required for the R&D community to effectively engage with biofoundries. Biofoundries provide the ability to do science that could not otherwise be done, and to deliver solutions that could not otherwise be developed; however, the research community needs to be trained on how to design appropriate experiments and what the costing structure is.
7. *Establish long-term funding mechanisms*. Biofoundries are expensive to establish and operate^3^; all current biofoundries in the academic context operate on a heavily subsidised model. Sustained funding requires sustained engagement with funding organisations and government to ensure recognition of the importance of biofoundries to individual countries’ pandemic response mechanisms.
8. *Engage and establish effective partnerships* with the public and private sector, such that biofoundry activities can be deployed where needed in emergencey scenarios, to enure maximal impact. Identify partners in the public health sector such that activities can be accredited and integrated into existing health delivery systems.

### Reporting summary

Further information on research design is available in the Nature Research Reporting Summary linked to this article.

## Supplementary information

Reporting Summary
